# Quality and Content Concordance of International Clinical Guidelines on Hypertensive Disorders of Pregnancy Using the AGREE II Instrument: An Updated Systematic Review

**DOI:** 10.3390/jcdd10070295

**Published:** 2023-07-11

**Authors:** Alessandra N. Bazzano, Alexandra Keenan, Sara Woltz, Advaith Subramanian, Onome Akpogheneta, Jorge Coronado Daza, Lydia A. L. Bazzano

**Affiliations:** 1 Department of Social, Behavioral, and Population Sciences, Tulane University School of Public Health and Tropical Medicine, New Orleans, LA 70112, USA; 2 Department of Epidemiology, Tulane University School of Public Health and Tropical Medicine, 1440 Canal St., New Orleans, LA 70112, USAlbazzano@tulane.edu (L.A.L.B.); 3 The Murphy Institute, School of Liberal Arts, Tulane University, New Orleans, LA 70118, USA; 4 Infectious Diseases Program, London School of Hygiene and Tropical Medicine, London WC1E 7HT, UK; 5 Department of Medicine, University of Cartagena, Cartagena 130014, Colombia

**Keywords:** hypertension, women’s health, pregnancy-induced hypertension, vascular diseases

## Abstract

Utilization of high-quality clinical practice guidelines has the potential to positively impact health outcomes. This study aimed to assess the quality and content concordance of national and international recommendations on hypertensive disorders of pregnancy (HDPs). Searches were conducted of the MEDLINE database and reference lists generated from national and international agencies. Covidence software was used for the management of the systematic review process, the Appraisal of Guidelines for Research and Evaluation II (AGREE II) tool was used to assess guidelines for quality, and three reviewers independently screened records. The research team identified and screened a total of 399 records of which 10 were deemed high quality. Guidelines were assessed and compared regarding the treatment, prevention, and categorization of disorders. The quality of guidelines varied across different domains, with significant variation in domain scores even within individual guidelines. Not all recommendations showed a high level of methodologic rigor, and the highest-rated guidelines were from the American Heart Association, the World Health Organization, and South Africa national guidelines. Classification of hypertension differed among the guidelines, particularly in defining chronic hypertension, severe hypertension, and preeclampsia. Prevention modalities varied across guidelines, with recommendations for aspirin, calcium supplementation, and against the use of certain approaches. Treatment modalities highlighted the importance of delivery as the definitive way to terminate hypertensive disorders of pregnancy, with other management strategies provided for symptom control. The variability in guidelines and consensus statements across different contexts may reflect regional differences in healthcare practices, available resources, and research evidence. There is potential to harmonize guidelines for HDP globally while considering the unique needs of individual countries. Where guidelines may be synthesized and condensed into an accessible format, doing so could improve their use in clinical decision-making.

## 1. Introduction

Hypertensive disorders of pregnancy (HDPs) are disorders of high blood pressure during pregnancy that occur in women who did not have hypertension prior to pregnancy, or preeclampsia in women with hypertension prior to pregnancy. Globally, 15% of all maternal deaths were caused by hypertensive disorders in 2017 [1], resulting in an estimated 29,375 maternal deaths and 1,872,699 DALY [1], therefore also increasing the risk of adverse outcomes for newborns. These disorders remain a significant cause of morbidity and mortality for mothers and offspring worldwide. Although maternal deaths from hypertensive disorders have declined globally by 30% between 1990 and 2019, the incidence of hypertensive disorders in pregnancy has risen by 10.9% during this period, from 16.3 to 18.1 million [2]. Increasing incidence trends have been observed in Eastern and Western Europe, Andean Latin America, North America, and the Caribbean [2]. In the United States, rates of hypertensive disorders in pregnancy are reported to have increased from 2.8% to 8.2% between 1989 and 2020 [3].

HDPs have been classified into four categories: gestational hypertension, preeclampsia/eclampsia, chronic hypertension, and chronic hypertension with superimposed preeclampsia/eclampsia [4,5]. In 2017, elevated blood pressure and stage 1 hypertension were also suggested as hypertensive disorders during pregnancy, thus emphasizing the growing burden of these disorders [6], and the need for blood pressure monitoring. Risk factors for chronic hypertension (high blood pressure before pregnancy or before 20 weeks of pregnancy) can be modified by changes in diet, physical activity, tobacco, alcohol consumption and weight management. Gestational hypertension usually occurs only during pregnancy and goes away within 12 weeks after birth, but it can lead to higher risks of future chronic hypertension after pregnancy. Preeclampsia can occur with the sudden development of high blood pressure, which can lead to life-threatening seizures (eclampsia) [7] for the mother and fetus. In the United States, preeclampsia occurs in 1 in 25 pregnancies [7], with higher rates among non-Hispanic black women; racial disparities have been identified both in the diagnosis and management of HDP [8].

Women with hypertensive disorders during pregnancy have elevated post-pregnancy risks of damage to the kidney, liver, brain and other organ systems [8,9]. Compared to women without preeclampsia, those with preeclampsia during pregnancy have an almost four times greater risk of developing chronic hypertension and are more than twice as likely to develop ischemic heart disease, venous blood clots and stroke post pregnancy [10]. Preeclampsia can lead to separation of the placenta from the uterus, stillbirth, preterm birth and pregnancy loss [8,9,10].

Neonatal and infant mortality risks are nearly doubled for the children of mothers with preeclampsia. However, a ‘protective’ association has also been reported for a subset of preterm or very low birth weight infants [11,12,13,14]. Among other complications for children born from mothers with preeclampsia, increased risk of cardiovascular defects, perinatal stroke and epilepsy have been reported [14,15,16,17,18,19,20,21,22,23]. Long-term health effects including elevated blood pressure in childhood and adolescence have also been reported for these children [14].

Effective classification, diagnosis and management of hypertensive disorders in pregnancy are crucial to reduce the risk of morbidity and mortality for mothers and offspring. However, globally, there are discrepancies in the classification, diagnosis and management of HDPs [5]. Policy-makers and health providers require robust guidelines to promote best practices for managing these disorders in order to improve the quality of healthcare and inform areas of future research. With guidelines developed for local, regional, national and international use, it is important to understand differences in guideline quality and rigor. By promoting the systematic examination of quality and consistency between varied clinical practice guidelines, concordance between guidelines can highlight best practices. The current systematic review aimed both to update a previous review [24] and to assess the quality and content concordance of contemporary clinical practice guidelines (CPG) on HDP from national and international sources.

## 2. Materials and Methods

We initiated a comprehensive guideline content and concordance review updating and extending the findings of a previous review. Study steps comprised a comprehensive search, formulation and application of selection criteria, assessment of quality using the Appraisal of Guidelines for Research and Evaluation (AGREE II) instrument [25], a compilation of results and analysis, per recommended practice. The principles of the PRISMA 2020 27-item checklist, an expanded checklist that details reporting recommendations, guided the review process. The PRISMA 2020 statement however was designed primarily for systematic reviews of studies that evaluate the effects of health interventions, rather than to review clinical guidelines, and this review was not registered as a systematic review for the same reason.

The MEDLINE database was searched for articles from 1 June 2014 to 31 December 2021, with an updated search conducted in December 2022. The starting date was selected to capture all guidelines that have been published since the previous systematic review [24]. Terms searched included *pregnancy*, *hypertension*, *pregnancy-induced hypertension*, and *hypertension in pregnancy* alone and in conjunction with guidelines and guidelines as a publication type. The full search strategy is outlined in Appendix A. Following a search of the MEDLINE database, a manual search was performed of guideline repositories and hand searched for guidelines in the following databases: Emergency Care Research Institute (ECRI) Guidelines Trust, Guidelines International Network’s website (GIN) and National Institute Health and Care Excellence’s website (NICE).

All databases and websites included in the search are listed here in alphabetical order: American College of Obstetricians and Gynecologists (ACOG), American Heart Association (AHA), Australian Government Department of Health, Canadian Journal of Cardiology, Canadian Medical Association (CMA), European Society of Cardiology (ESC) and the European Society of Hypertension (ESH) together, and ESC alone, as well as ECRI Guidelines, French Society of Hypertension (FSH), Guidelines International Network (GIN), Japanese Society of Hypertension (JSH), International Society of Hypertension (ISH), International Society for the Study of Hypertension (ISSHP), Malaysian Society of Hypertension (MSH), MEDLINE/PubMed, National Institute Health and Care Excellence Web site (NICE), NZ Ministry of Health (NZ), Pakistan Hypertension League, Philippine Society of Hypertension, Polish Societies of Hypertension, Saudi Hypertension Management Society, Scottish Intercollegiate Guidelines Network, Society of Obstetric Medicine of Australia and NZ (SOMANZ), the South African Medical Journal, Sudan Society of Hypertension (SSH), Taiwan Society of Cardiology together with the Taiwan Hypertension Society, and two separate guidelines from the World Health Organization (WHO).

Records identified through MEDLINE were screened using Covidence software to organize the review process [26]. Three reviewers independently screened records based on their titles and abstracts. Records were not selected for a full-text review if they were not a guideline, if they were not in English, if they were a duplicate, or if their scope did not include HDP. Disagreement between two reviewers was resolved with consensus from another author. Records identified through additional sources were assessed using the same criteria. With a goal to assess the management of HDP in general, guidelines were removed if they were limited to specific populations or if their scope was focused on specific conditions (e.g., guidelines covering only preeclampsia). Guidelines were only included if they were published within the specified dates 1 June 2014 to 31 December 2021, and if they were not primarily or entirely derived from other guidelines. All guidelines that met the criteria were extracted for a full-text review. Three authors independently reviewed selected documents.

To objectively assess guideline quality, we used the AGREE II instrument [25] assessed online. The AGREE II instrument consists of 23 items organized into 6 domains. Reviewers assessed each item and assigned a score from 1 (strongly disagree) to 7 (strongly agree). The overall quality of the guideline (1–7) and recommendations were also assessed. Three appraisers (AK, AS, SW) independently scored the guidelines using the AGREE II system. See Appendix A for full details.

Final domain scores (0% to 100%) were calculated based on the sum of ratings across all reviewers and scaled to include the maximum possible score as well as the difference between the maximum and minimum possible scores for that domain.

## 3. Results

**Figure 1:**
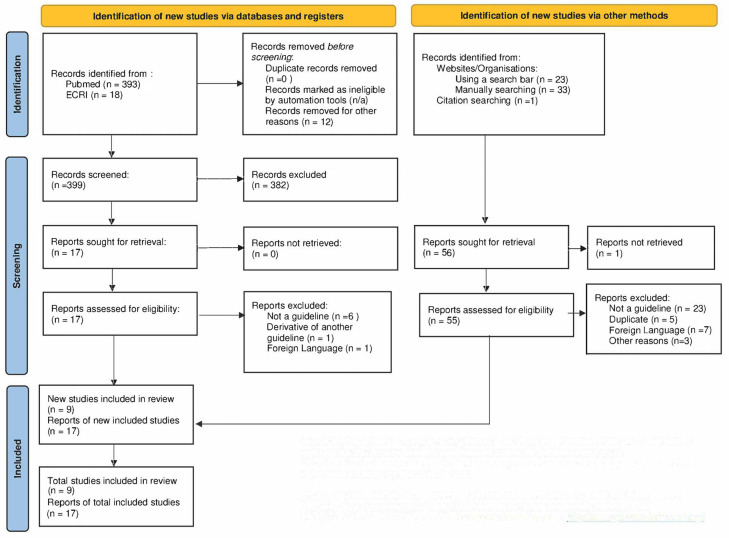
PRISMA flowchart.

Figure 1 provides a flowchart of the findings of the evaluation and review following the PRISMA standard. A total of 26 guidelines met the selection criteria for initial inclusion. These guidelines originated from several national and international sources; two guidelines were issued from one organization (focused on chronic vs. severe HDP) and were considered sufficiently distinct to be treated separately.

### 3.1. Appraisal of Guidelines and Consensus Statements

Using AGREE II domain scores, guidelines were deemed high quality based on overall domain score and rank among all guidelines screened full text. Average raw scores were used to rank order and 10 highly rated guidelines were extracted.

Table 1 shows the percentage of total points the 10 highest-rated guidelines received in each of the six quality domains that were assessed with the AGREE II tool. The quality of each CPG varied, and domain scores varied within guidelines. Scores for the scope and purpose ranged from 80–100%. Scores for stakeholder involvement ranged from 53% to 93%. Scores for rigor and development ranged from 77% to 100%. Scores for the ‘clarity of presentation’ domain ranged from 88% to 100%. Both the WHO Non-Severe and Severe Hypertension guidelines received 88% in this domain, while the AHA, CMA and ESH all received 100%. In the domain ‘applicability’, scores had a wide range, from 67% to 100%, and in the domain ‘editorial independence’, scores ranged slightly less from 71% to 100%. Seven of the ten guidelines received all points in the ‘editorial independence’ domain. Across all domains, the AHA scored the highest of the ten, receiving 100% of the points in every domain except for ‘stakeholder involvement’.

### 3.2. Classification of Hypertension

The classification of hypertension by high-quality CPGs is illustrated in Table 2. The GPCs from the FSH, NZ, NICE and SA included the definition of hypertension prior to 20 weeks of gestation, the AHA, CMA, ESC and WHO did not define chronic hypertension, and MSH defined it as persisting past 12 weeks. Severe hypertension was defined as BP ≥ 160/110 mmHg (N = 6) for all CPGs with the exception of ≥170/110 mmHg defined by the MSH, and WHO did not provide a definition. Preeclampsia was defined by hypertension after 20 weeks and proteinuria among all guidelines with the exception of the AHA and ASC, whose definitions included only proteinuria and WHO which did not provide a definition. Preeclampsia is a multi-system syndrome affecting the fetus and/or one or more organ systems (AHA). The organ systems include hepatic renal hematological pulmonary and neurologic. Fetal placental complications include fetal growth restriction (CMA, FSH, MSH, NZ and NICE), oligohydramnios (FSH and MSH), and placental abruption (FSH, NZ and NICE). Proteinuria definitions varied, including >300 mg/24 h from a urine sample (CMA, FSH, and MSH) ≥30 mg/mmol protein: creatine ratio on a spot urine protein test (ESC, FSH, NICE, NZ and MSH), ≥2 on a dipstick test (ESC, MSH and NZ), albumin: creatinine ratio of ≥8 mg/mmol to be used as an alternative to protein: creatinine ratio or dipstick test >1 (NICE). The WHO, AHA and SA CPGs did not define proteinuria although the SA did recommend testing with a clean catch urine specimen without white blood cells or nitrates. Severe preeclampsia was defined as systolic blood pressure of ≥160 mmHg and diastolic blood pressure of ≥110 mmHg (AHA, CMA, ESC, FSH, MSH, NZ, NICE, SA and WHO), systolic blood pressure of ≥180 mmHg and diastolic blood pressure of ≥120 mmHg (MSH) and was not defined by the WHO CPG. HELLP syndrome was defined in all 6 CPGs as hemolysis, elevated liver enzymes and low platelet count. Eclampsia was defined as new onset of seizures associated with preeclampsia (NZ, NICE and FSH) and may occur before, during or after labor (NZ). White coat hypertension was defined by untreated hypertension SBP >130 mmHg, but <160 mmHg or DBP >80 mmHg, but <100 mm Hg or suspected white coat hypertension. The AHA recommended screening for white coat hypertension with either daytime 24 h ambulatory blood pressure measure (ABPM) or home blood pressure monitoring (HBPM). NZ did not define when screening is required but recommended 24 h ABPM and HBPM. Other CPGs did not define white coat hypertension.

### 3.3. Prevention Modalities

Various prevention modalities were described by the CPGs, including pharmaceutical and non-pharmaceutical options.

#### 3.3.1. Aspirin

Low-dose acetylsalicylic acid use for preeclampsia prevention in high-risk women was recommended in 10 of 12 CPGs. However, dosage and timing varied among the guidelines. Dosages ranged from 75 to 162 mg depending on the CPG. Women considered high risk were those who have a personal or family history of HDPs, chronic medical disease and/or abnormal uterine artery Doppler before 24 weeks gestation. Recommendations regarding the timing of initiation of aspirin varied by CPG. Most guidelines stated therapy should be started before 16 weeks of gestation, but this recommendation ranged from between 12 to 20 weeks of gestation. Most guidelines recommended therapy continue until delivery.

#### 3.3.2. Calcium

Five CPGs recommended calcium supplementation (ESC, MOH, NZ, SA and WHO). Most guidelines recommended low-dose calcium supplementation for women with low dietary intake of calcium (<600 mg/day), especially among women at high risk of developing preeclampsia. Calcium dosages ranged from 1.0 g to 2.0 g/day. The SA guideline stated calcium supplementation should be given to all pregnant women, regardless of population risk factors and dietary calcium intake.

#### 3.3.3. Vitamins C and E

Five CPGs recommend against prophylactic antioxidant therapy with vitamins C and E (ESC, MOH, NZ, NICE and WHO) due to its association with low birth weight and other adverse perinatal outcomes.

#### 3.3.4. Folic Acid

Two CPGs mentioned folic acid. NZ recommended folic acid for all pregnancies to reduce the risk of spina bifida and to promote normal brain development. NICE mentioned folic acid supplementation as a means of preventing hypertensive disorders during pregnancy, without discussion of potential benefits.

#### 3.3.5. Low Molecular Weight Heparin

Two CPGs recommended against the use of low molecular weight heparin (LMWH) for HDPs (FSH and NICE).

#### 3.3.6. Other Therapies

The 10 highest-ranked guidelines recommended against therapies including nitric oxide donors (France and NICE), progesterone (NICE), thiazide diuretics (NICE and WHO) and bed rest (NZ, NICE and WHO). Several CPGs did not recommend the following supplements for the prevention of hypertensive pregnancy disorders: fish oil (MOH, NZ and NICE), garlic (MOH, NZ and NICE), magnesium (NZ, NICE) and Vitamin D (MOH, WHO). Three CPGs made recommendations against dietary salt restriction (NZ, NICE and WHO).

Full details of prevention modalities extracted from guidelines are outlined in Table 3 below.

### 3.4. Treatment Modalities

#### 3.4.1. Delivery

Timing of birth was included in six of the highest-ranking CPGs. All guidelines recommended delivery as the only definitive way to resolve HDPs, but recommendations were provided for the management of symptoms. Delivery was recommended at ≥37 weeks gestation for women with gestational hypertension, chronic hypertension, or moderate preeclampsia. Four CPGs recommended delivery at >34 weeks for women with severe preeclampsia and adopted an expectant approach for women <34 weeks. However, this recommendation was made among all CPGs to expedite delivery in preeclamptic patients with deteriorating maternal–fetal conditions by all CPGs. Timing of birth should be based on whether the woman and baby are at risk of adverse outcomes if pregnancy is prolonged.

A full description of treatment modalities recommended by CPGs is presented in Table 4.

#### 3.4.2. Bed Rest

Two CPGs recommended against bed rest for gestational hypertension (NICE) and for the prevention of hypertensive disorders in pregnancy (WHO). One CPG recommended against strict bed rest for improving pregnancy outcomes in women with hypertension (WHO).

#### 3.4.3. Antihypertensives

Methyldopa and labetalol were considered first-line treatment options by all highly-rated guidelines. Calcium antagonists, specifically nifedipine, were cited as a first-line treatment option by nine CPGs. Hydralazine was not considered a drug of choice, but it was recommended when other treatment regimens have failed to achieve adequate BP control by some guidelines (ESC, MSH and MOH). Sodium nitroprusside was considered a last resort drug treatment by two guidelines (ESC and WHO) due to prolonged treatment being associated with an increased risk of fetal cyanide poisoning. When preeclampsia was associated with pulmonary edema, nitroglycerin, given as an intravenous infusion, was recommended by one guideline (ESC).

ACE inhibitors and angiotensin receptor blockers were contraindicated in pregnancy by nine of 10 CPGs (SA made no recommendation either for or against). Direct renin inhibitors were also not recommended (AHA, ESC, FSH and MSH).

#### 3.4.4. Corticosteroids

To reduce the risk of neonatal death, respiratory distress syndrome, cerebrovascular hemorrhage, necrotizing enterocolitis, respiratory support and intensive care unit (ICU) admission, administration of corticosteroids were recommended in cases of severe hypertension, and preeclampsia with severe features before 34 weeks (FSH, NICE, NZ and SA).

#### 3.4.5. Intravenous Fluid Maintenance

Administration of crystalloid fluid was recommended before or when administering the first IV hydralazine dose (NICE, NZ). One CPG recommended strict fluid monitoring in women with severe preeclampsia or eclampsia intrapartum and postpartum (NZ). In women with severe preeclampsia, recommendations were made to limit total fluids to 80 mL/h (NICE) or 80–85 mL/h (NZ) to reduce the risk of pulmonary oedema.

#### 3.4.6. Magnesium Sulfate

Four CPGs recommended magnesium sulfate for the prevention and treatment of eclampsia as the drug of choice over other anticonvulsants (FSH, NICE, SA and WHO). Four guidelines recommended the use of magnesium sulfate for the treatment of preeclampsia with severe features (FSH, NICE, SA and WHO). A loading dose of 4 g was recommended intravenously over 5–15 min (NICE) and over 20 min (SA). One CPG specified 1 g/h should be administered for 24 h after the last eclamptic episode, noting recurrent episodes should be treated with a further dose of 2 to 4 g given intravenously over 5 to 15 min (NICE). If magnesium sulfate is administered for more than 5 to 7 days in pregnancy or is repeatedly used, fetal surveillance is recommended because its prolonged use has been associated with skeletal adverse effects and hypocalcemia and hypermagnesemia in neonates (NICE). One CPG-specified magnesium sulfate should not be given concomitantly with calcium channel blockers (ESC).

#### 3.4.7. Fetal Surveillance

Fetal surveillance recommendations varied among CPGs and differed in modality and frequency depending on the classification of HDPs.

Early dating ultrasound (NICE, FSH and SA), ultrasound for fetal growth (NICE, FSH and SA), fetal heart auscultation, amniotic fluid volume and umbilical artery doppler velocimetry were all considered acceptable modalities for fetal surveillance. Cardiotocography was recommended if a cardiac anomaly was detected by two guidelines (NICE, MSH).

## 4. Discussion

CPG content and concordance covering the diagnostic approach and management of HDPs were assessed using the AGREE II instrument. Guidelines came from a range of countries, professional associations and international agencies. AHA guidelines scored highest using the AGREE II instrument [25] across all domains, followed by the World Health Organization guideline for non-severe hypertension, and SA. Three guidelines scored higher compared to others under the domain rigor of development, including AHA and WHO Non-severe Hypertension guidelines, as well the WHO Severe Hypertension guideline. Notably, guidelines from SA did not score highly in the rigor of development, with Canadian and NZ guidelines scoring higher in that category but lower overall.

In relation to our prior review [24], several differences have been noted. First, a greater number of guidelines were assessed as scoring highly on AGREE II ratings. For example, both the WHO guidelines for acute and chronic hypertension [27,28] were not included in the prior review, and in the current review were highly rated per AGREE II. Several guidelines that had been included in the prior review, e.g., SOMANZ and ACOG guidelines [29,30], were not ranked among the 10 highest scoring in the current review and thus recommendations were not extracted (see Appendix A). Only NICE [31] and ESC [32] guidelines from the prior review were included in the current review as highly rated.

Also in contrast to the prior review, the current review included guidelines from a wider geographic range, including highly rated guidance from sub-Saharan Africa [33], Asia [34], Australasia [35], Europe [32,36] and North America [5,37].

Our findings stand in contrast to a review of CPGs from 2009–2019 [38]. In the review by Scott et al., two authors reviewed CPGs, deemed 15 guidelines “clinically useful” and abstracted recommendations from these. Where the review by Scott and colleagues focused on treatment recommendations, we applied a broader approach to quality in the current review. Another recent study comparing international guidelines for the prevention, diagnosis and management of HDPs was published in 2020 [39]. The study by Sinkey et al. did include some of the same guidelines as those included in the Scott et al. review and the current review, including, for example WHO and ESC; however, no rationale nor methodology was provided for the selection of guidelines reviewed. Similarly, the Sinkey et al. study did not utilize a structured validated tool like AGREE II for comparison of guideline content and concordance, nor was a systematic review process described.

Strengths of the current study include the evaluation of each guideline by three independent raters, library scientist input, a comprehensive search strategy with the use of multiple databases and the use of a structured, validated quality rating tool. Limitations of the study were the inclusion of only English language guidelines. Due to the inability to review guidelines in other languages, this may have resulted in the exclusion of potentially relevant guidelines designed for use in non-English speaking countries. Finally, the review centered on guidelines of broader scope which may have resulted in failure to identify guidelines that, though of narrow scope, may have been pertinent to recommendations.

## 5. Conclusions

Multiple international and national guidelines exist on the diagnosis, prevention and management of HDP. Many of these guidelines concur in their recommendations regarding the prevention and treatment of HDP, severe hypertension, preterm preeclampsia and eclampsia, and yet, there is considerable variability. The lack of clear consensus and concordance across international guidelines poses the challenge of the inaccessibility of recommendations for clinical practice.

The variability in guidelines and consensus statements across different contexts may reflect regional differences in healthcare practices, available resources, and research evidence. There is potential to harmonize guidelines for HDP globally while considering the unique needs of individual countries. Where guidelines may be synthesized and condensed into an accessible format, doing so could improve their use in clinical decision-making.

## Figures and Tables

**Table 1:** Individual AGREE II domain scores for top-ranked CPG represented as a percentage (%) score.

Domain	AHA	WHO Non-Severe	SA	WHO Severe	NZ	NICE	CMA	MSH	ESC	FSH
Scope and Purpose	100	96	93	96	100	90	100	80	92	98
Stakeholder Involvement	93	90	80	79	88	80	87	80	53	74
Rigor of Development	100	97	77	98	87	86	94	85	89	78
Clarity of Presentation	100	88	96	88	96	95	100	93	100	95
Applicability	100	96	84	92	85	73	67	77	69	45
Editorial Independence	100	100	100	100	100	71	100	85	76	100

**Table 2:** Classification of hypertension by clinical practice guideline.

		AHA	CMA	ESC	FSH	MSH	NZ	NICE	SA	WHO Severe and Non-Severe
Chronic hypertension	Persists past 12 weeks					*				
	Persists past 6 weeks									
	Prior to 20 weeks of gestation				x		x	x	x	
Severe hypertension	180/120					†				
	170/110									
	160/110	x	x	x	x		x	x	x	
White coat hypertension		x	x	x	x	x	x		x	x
Masked hypertension		x			x	x				x
Gestational hypertension			x	x	x	x	x	x	x	x
Preeclampsia	Hypertension after 20 weeks		x		x	x	x	x	x	
	Protenuria	x	x	x	x	x	x	x	x	
Sequelae of preeclampsia	Liver dysfunction		x	x	x	x	x	x	x	
	Renal dysfunction		x	x	x	x	x	x	x	
	Hematological dysfunction				x	x	x	x	x	
	Pulmonary edema			x	x	x	x	x	x	
	Visual, neurologic or cerebral disturbances				x	x	x	x		
	Fetal growth restriction		x		x	x	x	x		
	Oligohydramnios				x	x				
	Placental abruption				x		x	x		
	Absent or reversed end-diastolic flow									
	Abnormal fetal heart rate									
Superimposed Preeclampsia			x	x	x	x	x			
Eclampsia		x		x	x		x	x	x	x
HELLP Syndrome			x		x		x	x	x	x

* Chronic hypertension is diagnosed when hypertension and/or proteinuria persist after three months postpartum; † >180/120; x: definition provided in the guideline.

**Table 3:** Prevention modalities by CPG.

	AHA	CMJ	ESC	FSH	MSH	NZ	NICE	SA	WHO *
Aspirin			+	+	+	+	+	+	+
Calcium			+		+	+		+	+
Vitamin C + E			X	X	X	X	−		X
LMWH				X			X		
Fish oil					−	−	−		
Folic acid						−	−		
Nitric oxide donors				X			X		
Progesterone							X		
Magnesium						−	−		
Salt restriction						X	−		X
Caloric restriction									
Thiazide diuretics							X		X
Bed rest						X			X
Physical exercise				X					

* All WHO recommendations came from their recommendations for prevention and treatment of preeclampsia and eclampsia that were cited in their drug treatment for severe hypertension in pregnancy guideline; X: recommended against, + = recommended by guideline, −: not recommended for or against.

**Table 4:** Treatment options endorsed for HDP by CPG.

Treatment	AHA	CMJ	ESC	FSH	MSH	NZ	NICE	SA	WHO
Antihypertensive therapy									
Methyldopa	**+**	**+**	**+**	**+**	**+**	**+**	**+**	**+**	**+**
Beta-blocker		**+**	**+** Avoid atenolol	**+** Avoid atenolol	+ Not atenolol	**+**		+(IV labetalol for severe)	**+**
CCB	**+**	**+**	**+**	**+**	**+**	**+**	**+**	**+**	+ (for severe)
Diuretics		**+**	**X**	**X**	**X**		**X**		
ACE inhibitors	**X**	**X**	**X**	**X**	**X**	**X**	**X**		**X**
ARBs	**X**	**X**	**X**	**X**	**X**	**X**	**X**		**X**
Direct renin inhibitors	**X**		**X**	**X**	**X**				
Centrally acting alpha agonist (Clonidine)		**+ (2nd line)**							
Vasodilators (hydralazine)		**+**	**−** used when other drug regimens fail (severe)		**+**	**+** (for severe)	**+** (for severe)	**+** (for severe)	**+**
**Antenatal corticosteroids**				**+**		+	**+**	**+**	
**Nitroglycerin**			**+**						
**Sodium nitroprusside**			**−** (Severe)						X
**Magnesium sulfate**									
For severe preeclampsia and/or eclampsia			**+**	**+**	**+**	**+**	**+**	**+**	+ *
**Timing of Delivery**									
Gestational hypertension			37 weeks			37–40 weeks	>37 weeks	38–40 weeks	
Preeclampsia			37 weeks	37 weeks		<37 weeks, adopt expectant approach; >37 weeks	>34 weeks and 34–36 weeks continue surveillance; >37 weeks initiate birth	>34 weeks delivery should be offered	
Severe preeclampsia			Expediate delivery	Discuss termination if onset is <24 weeks; if<34 weeks deliver		<34 weeks adopt expectant approach >34 weeks deliver	<34 weeks	28–33 weeks Initiate delivery after 48 h if woman is stable; 34 weeks deliver	Immediate

X: recommended against, + = recommended by guideline, −: not recommended for or against; * WHO recommendation came from their recommendations for prevention and treatment of preeclampsia and eclampsia that was cited in their drug treatment for severe hypertension in pregnancy guideline.

## Data Availability

All data used are publicly available.

## Appendix A

**Table A1:** Additional clinical practice guideline domain scores using the AGREE-II instrument.

Domain	ESC/ESH	Australia	IS—H	Japanese SOH	Philippines	Taiwan	Poland	ISSHP
Scope and Purpose	100%	100%	100%	100%	100%	79%	87%	100%
Stakeholder Involvement	62%	81%	70%	70%	79%	67%	52%	62%
Rigor of Development	63%	73%	51%	80%	65%	76%	57%	51%
Clarity of Presentation	100%	94%	92%	89%	92%	90%	100%	94%
Applicability	73%	96%	76%	65%	55%	58%	67%	64%
Editorial Independence	76%	100%	64%	48%	67%	71%	81%	57%

**Table A2:** Additional clinical practice guideline domain scores using the AGREE-II instrument.

Domain	ACOG	Saudi	Pakistan	Sudan	Norway	Portuguese	SOMANZ
Scope and Purpose	95%	94%	83%	94%	41%	79%	71%
Stakeholder Involvement	46%	76%	62%	78%	54%	38%	25%
Rigor of Development	56%	54%	39%	32%	54%	25%	32%
Clarity of Presentation	90%	81%	86%	81%	94%	90%	60%
Applicability	29%	55%	58%	51%	31%	43%	44%
Editorial Independence	57%	14%	29%	14%	29%	14%	14%

AGREE-II, Appraisal of Guidelines for Research and Evaluation II; ESC/EHS, European Society of Cardiology/European Society of Hypertension; Australia, Australia Government Department of Health; ISH, International Society of Hypertension; Japanese SOH, Japanese Society of Hypertension; Philippines, *The Journal of Clinical Hypertension*; Taiwan, *Journal of the Chinese Medical Association*; Poland, Polish Society of Hypertension/Polish Cardiac Society/Polish Society of Gynecologists and Obstetricians; ISSHP, International Society For The Study Of Hypertension In Pregnancy; ACOG, The American College of Obstetricians and Gynecologists; Saudi, Saudi Hypertension Management Society; Pakistan, Pakistan Hypertension League; Sudan, Sudan Society of Hypertension; Norway, *European Journal of Obstetrics & Gynecology and Reproductive Biology;* Portuguese, Arquivos Brasileiros de Cardiologia; SOMANZ, Society of Obstetric Medicine Of Australia and NZ.

**Table A3:** AGREE II Raw scores.

Domain	AHA	WHO Non-Severe	SA	WHO Severe	NZ	NICE	CMJ	MSH	ESC	FSH
Scope and Purpose	63 (100.00%)	61 (96.83%)	59 (93.65%)	61 (96.83%)	63 (100.00%)	57 (90.48%)	63 (100.00%)	51 (80.95%)	58 (92.06%)	62 (98.41%)
Stakeholder Involvement	59 (93.65%)	57 (90.48%)	51 (80.95%)	50 (79.37%)	56 (88.89%)	51 (80.95%)	55 (87.30%)	51 (80.95%)	34 (53.97%)	47 (74.60%)
Rigor of Development	168 (100.00%)	164 (97.62%)	131 (77.98%)	165 (98.21%)	147 (87.50%)	145 (86.31%)	158 (94.05%)	144 (85.71%)	151 (89.88%)	132 (78.57%)
Clarity of Presentation	63 (100.00%)	56 (88.89%)	61 (96.83%)	56 (88.89%)	61 (96.83%)	60 (95.24%)	63 (100.00%)	59 (93.65%)	63 (100.00%)	60 (95.24%)
Applicability	84 (100.00%)	81 (96.43%)	71 (84.52%)	78 (92.86%)	72 (85.71%)	62 (73.81%)	57 (67.86%)	65 (77.38%)	58 (69.05%)	38 (45.24%)
Editorial Independence	42 (100.00%)	42 (100.00%)	42 (100.00%)	42 (100.00%)	42 (100.00%)	30 (71.43%)	42 (100.00%)	36 (85.71%)	32 (76.19%)	42 (100.00%)
Overall	21 (100.00%)	20 (95.24%)	20 (95.24%)	19 (90.48%)	19 (90.48%)	19 (90.48%)	18 (85.71%)	18 (85.71%)	18 (85.71%)	18 (85.71%)
